# Fibroadenoma in axillary accessory breast mimicking carcinoma of unknown primary; a case report with literature review

**DOI:** 10.1016/j.amsu.2021.103179

**Published:** 2021-12-14

**Authors:** Lana R.A. Pshtiwan, Zuhair D. Hammood, Abdulwahid M. Salih, Sanaa O. Karim, Bakhan Sharif Ali, Fahmi H. kakamad, Razhan K. Ali

**Affiliations:** a Smart Health Tower, Madam Mitterrand Street, Sulaimani, Kurdistan, Iraq; b College of Medicine, University of Sulaimani, Madam Mitterrand Street, Sulaimani, Kurdistan, Iraq; c College of Nursing, University of Sulaimani, Madam Mitterrand Street, Sulaimani, Kurdistan, Iraq; d Kscien Organization, Hamdi Str, Azadi Mall, Sulaimani, Kurdistan, Iraq; e Shar Hospital, Sulaimani, Kurdistan, Iraq

**Keywords:** Accessory breast, Ectopic breast, Axilla, Fibroadenoma, Carcinoma of unknown primary

## Abstract

**Introduction:**

Accessory breast (AB) is extra and ectopic breast tissue. Fibroadenoma (FA) in AB is a rare finding. This study aims to present a case of FA in axillary AB mimicking carcinoma of unknown primary (CUP).

**Case report:**

A 38-year-old female presented with a mass in her right axilla. She had a mass in her right breast for 5 years. She previously had a left breast lumpectomy for a benign condition. The lump in her axilla was palpable and hard. Ultrasound showed an oval lymph node in the level I axilla (12*6mm) with blurred and unclear fatty hilum, suspicious for malignancy. Magnetic resonance imaging revealed an oval lesion (12*7mm) in the level I axilla with no fatty hilum and with heterogeneous enhancements, suggesting abnormal nodes. Fine needle aspiration of the axillary mass suspected CUP. But core biopsy resembled FA. Both masses in the right axilla and breast were surgically excised. Histopathology confirmed FA in both masses.

**Discussion:**

Although pathologies in AB are uncommon, it is still susceptible to the same malignant and benign transformations that are found in normal breasts. The axilla is the most frequent location for FA in AB and often affects young women. Imaging techniques can be inconclusive and only histopathology can conclude a definitive diagnosis.

**Conclusion:**

FA in axillary AB is a rare condition that causes a diagnostic dilemma as it can be mistaken for other benign or malignant pathologies.

## Highlights

• Accessory breast is having extra and ectopic breast tissue.
• Fibroadenoma in the accessory breast is a very rare finding.
• It can be mistaken for other malignant pathologies.
• This report presents a case of fibroadenoma in axillary accessory breast mimicking carcinoma of unknown primary.

## 1 Introduction

Accessory breast (AB) is defined as the presence of extra and ectopic breast tissue. It is found anywhere along the milk lines, with axillary localization being most frequently reported [1]. AB is thought to be an embryonic mammary tissue remnant that can occur with or without the nipple and areola [2,3]. It is associated with an incidence of 1–6% in women which is much higher than in males (1.68%) [1,4]. Even though pathologic conditions in AB are considered highly uncommon, they can still occur as AB tissue has a similar stroma and epithelium to that of normal breast tissue. Hence, the diagnosis of AB is significant as it is susceptible to the same pathologic changes occurring in normal breasts [5,6]. The diagnosis of AP that lacks nipple and areola is challenging post-operatively, and the most common differential diagnosis for the condition can include apocrine gland tumor, lipoma, lymphoma, and lymphadenopathy [2,7]. Fibroadenoma (FA) is a well-described and frequently benign tumor of the breast - more commonly found in young women [8]. The occurrence of FA in AB is considered a very rare finding [9]. On rare occasions, AB has been observed to mimic malignancy [10,11].

This study aims to present a rare case of FA in axillary AB mimicking carcinoma of unknown primary (CUP), with a brief review of the literature. SCARE 2020 guidelines were taken into consideration in the writing of the current paper [12].

## 2 Case presentation

Patient information: A 38-year-old female presented with a mass in her right axilla. It was accidently found while visiting clinic for her routine follow-up of a mass in her right breast which she had for 5 years. She was G2P2 and lactated for 3 years. She did not have any chronic illnesses. Her surgical history included a left breast lumpectomy for a benign mass that was performed 20 years prior to this report. Her family lacked a history of cancer.

Clinical findings: The patient's right breast mass was palpable, firm, freely mobile, with mild tenderness. The lump in her axillary area was palpable and hard.

**Fig. 1:**
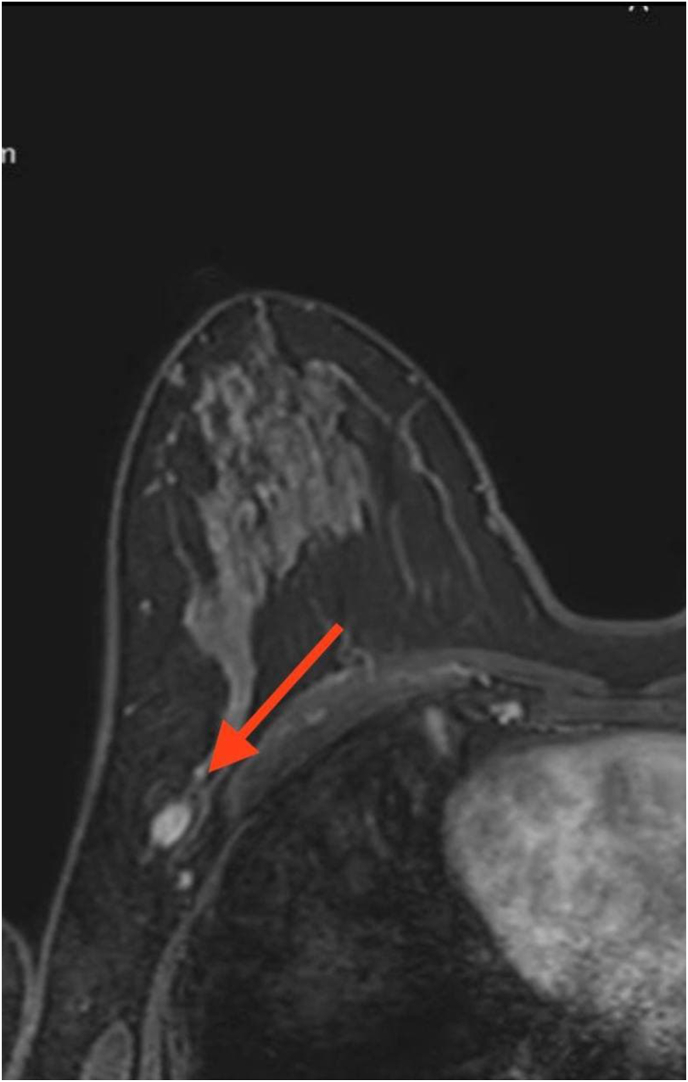
Axial T1 magnetic resonance imaging (MRI) with contrast-fat sat: show an oval circumscribed (mass) homogeneous enhancement with thin linear central hypo intense signal simulating axillary node.

Diagnostic approach: The patient's routine laboratory findings were normal. Ultrasound (US) examination revealed a benign mass (U2) measuring 9*6 mm in the left breast at 4 o'clock position in a middle depth (35 mm), two circumcised small foci (U2) of about 5 mm in the right breast at a middle depth with no suspicious features, and an oval lymph node in the level I axilla measuring 12*6 mm with blurred and unclear fatty hilum, suspicious for malignancy. Magnetic resonance imaging (MRI) showed a 13 mm circumcised mass in the left breast 14 mm from the skin at 4 o'clock direction, two small foci (5 and 4 mm) in the right breast with homogeneous enhancements which was not restricted on DWI/ADC with type I (slow raising and persistent delayed phase benign findings), and an oval lesion measuring 12*7 mm in the level I axilla with no fatty hilum and low signal on T1WI, an intermediate signal in T2WI, high signal on Dixon water, with heterogeneous enhancements suggesting abnormal nodes (Fig. 1). Fine needle aspiration (FNA) of the axillary mass suspected malignancy of CUP type. Core needle biopsy was then advised which revealed benign non-proliferative fibrocystic changes with fibroadenomatoid hyperplasia in the right breast mass and FA in the right axillary lymph node.

Therapeutic intervention: Under general anesthesia, right breast lumpectomy and right axillary mass excision were performed to remove the masses. Post-operative histopathology examination showed two fibroadenomas with proliferative fibrocystic changes in the right breast and a fibroadenoma with benign ectopic breast tissue in the right axilla.

Follow-up and outcome: The operation was uneventful and the patient was discharged in good health.

## 3 Discussion

AB is a relatively common condition amongst the female population (1–6%). Other terms for this condition include polymastia, supernumerary breast, and ectopic breast [1]. The newest classification of ectopic breast tissues includes eight subtypes (I-VIII) which are based on the presence of any or combination of the nipple, areola, and glandular tissue [13]. Even though pathologic conditions in AB are uncommon, it is still susceptible to the same malignant and benign transformations that are found in normal breast tissues, including cystosarcoma phyllodes, FA, carcinoma, fibrosis, mastopathy, and inflammation [5,6]. Breast cancer is the most common pathology found in AB followed by mastopathy - with FA rarely being observed in the literature [2].

During embryonic development, at the 6th week of gestation, ectodermal thickenings form mammary ridges that extend from the axilla to the groin. The pectoral region of this ridge will form normal breasts and the rest will undergo atrophy [14]. Regarding the formation of AB, two theories have been put forward. One theory postulates that during embryonic development the failure to atrophy in some regions along the mammary ridge leads to the development of AB. The other theory suggests that AB develops from the modification of apocrine sweat glands [15]. Hence, AB tissues are considered as embryonic mammary tissue remnants which are observed along the milk line - with the axillary region being the most frequent localization [2]. However, reports on vulval, perineal, and facial localizations have also been reported [14]. FA in AB is a very rare entity with only a few cases being reported in the literature [4].

FA is a well-circumscribed, benign, mobile, palpable, and painless tumor that is more prevalent among the female population of under 30 years [6,16]. However, in some cases of FA in AB, the mass was not palpable [1]. In the current case, FA was palpable. Generally, FA in AB is sporadic with only a small portion being familial (6%) [4]. FA in AB has a much higher incidence rate in females than in males [9]. Most reports of FA in AB have been observed in the axilla - with the vulva being the second most frequent site [2]. The axilla was also the involved site in this study. The condition usually starts developing when there is a hormonal stimulation, such as during lactation, pregnancy, or puberty, and tends to be asymptomatic [5,17]. According to Laporte et al., the mean age of patients diagnosed with FA in axillary AB is 33 years with most of them being in the 3rd and 4th decade of life [7]. However, Yilmaz and associates reported a 13-year-old case [8]. Gentile et al. presented a 58-year-old patient [18]. The patient included in this study was 38 years old. Most reported cases of FA in AB were also associated with FA in the normal breasts [2] which was also the case in this study. An association has been reported between AB and renal malformations; however, it has stayed controversial [14]. No renal or urological abnormalities were noted in this case.

In the absence of nipple and areola in the AB, clinical diagnosis can be highly challenging [17]. Reportedly, only in 23.2% of the cases, correct clinical diagnosis has been achieved, and in most instances, the condition was mistaken for lipoma, lymphadenopathy, follicular cyst, or hidradenitis [6]. In the diagnosis of FA in AB the same approach as in normal breast can be undertaken [13]. US, MRI, mammography, FNA, and core biopsy can be used in its pre-operative diagnosis [5,9]. However, imaging techniques can be inconclusive and only histopathology can conclude a definitive diagnosis [1]. FA in AB can also be mistaken for malignancy. Sawa et al. presented a case that was pre-operatively considered as CUP and metastases from breast cancer that was histologically confirmed to be FA in axillary AB [2]. In this study, FA in axillary AB mimicked CUP. Hence, when a mass is noticed in the axillary region, FA of BA should also be considered as a differential diagnosis [19].

For the treatment of these cases, complete surgical excision is recommended - especially if the mass is symptomatic, causes psychological distress due to cosmetic concerns, or there is doubt regarding the diagnosis [5,20].

In conclusion, FA in axillary AB is a rare condition that causes a diagnostic dilemma as it can be mistaken for other benign or malignant pathologies. It most often affects adult females in the 3rd and 4th decade of life. Imaging modalities can sometimes be inconclusive in their diagnosis; hence, histopathology is often required. Complete surgical excision of the mass and AB tissue is recommended.

## Funding

None is found.

## Ethical approval

Not required for case report.

## Consent

Consent has been taken from the patients and the family of the patients.

## Author contribution

Abdulwahid M. Salh: major contribution of the idea, literature review, final approval of the manuscript.
Zuhair D. Hammood: Surgeon performing the operation, final approval of the manuscript.
Fahmi H. Kakamad: literature review, writing the manuscript, final approval of the manuscript.
Bakhan Sharif Ali, Razhan K. Ali, Sanaa O. Karim, Lana R.A. Pshtiwan: literature review, final approval of the manuscript.

## Registration of research studies

1. Chinese Clinical Trial Registry
2. ChiCTR2100047387
3. Chinese Clinical Trial Register (ChiCTR) - The world health organization international clinical trials registered organization registered platform

## Guarantor

Fahmi Hussein Kakamad is Guarantor of this submission.

## Declaration of competing interest

None to be declared.
